# Prevention and management of radiotherapy-related toxicities in gynecological malignancies. Position paper on behalf of AIRO (Italian Association of Radiotherapy and Clinical Oncology)

**DOI:** 10.1007/s11547-024-01844-5

**Published:** 2024-08-28

**Authors:** Elisabetta Perrucci, Gabriella Macchia, Annamaria Cerrotta, Angela Damiana Andrulli, Rosa Autorino, Amelia Barcellini, Maura Campitelli, Giulia Corrao, Sara Costantini, Vitaliana De Sanctis, Jacopo Di Muzio, Valeria Epifani, Patrizia Ferrazza, Andrei Fodor, Elisabetta Garibaldi, Concetta Laliscia, Roberta Lazzari, Elena Magri, Cristina Mariucci, Maria Paola Pace, Brigida Pappalardi, Alice Pastorino, Federica Piccolo, Claudio Scoglio, Alessia Surgo, Francesca Titone, Francesca Tortoreto, Francesca De Felice, Cynthia Aristei

**Affiliations:** 1 Radiation Oncology Section, Perugia General Hospital, Perugia, Italy; 2 Radiation Oncology Unit, Responsible Research Hospital, Campobasso, Italy; 3 Radiotherapy Unit, Fondazione IRCCS, Istituto Nazionale dei Tumori, Milan, Italy; 4 Radiotherapy Unit, Azienda Ospedaliera San Giovanni Addolorata, Rome, Italy; 5 UOC di Radioterapia, Dipartimento di Scienze Radiologiche, Radioterapiche ed Ematologiche, Fondazione Policlinico Universitario Agostino Gemelli IRCCS, Rome, Italy; 6 Radiation Oncology Unit, Clinical Department, CNAO National Center for Oncological Hadrontherapy, Pavia, Italy; 7 Department of Internal Medicine and Medical Therapy, University of Pavia, Pavia, Italy; 8 Department of Radiotherapy, IEO European Institute of Oncology IRCCS, Milan, Italy; 9 Radiation Oncology Centre, Santa Maria Hospital, Terni, Italy; 10 Radiotherapy Oncology, Department of Medicine, Surgery and Translational Medicine, St. Andrea Hospital, Sapienza University of Rome, Rome, Italy; 11 Dipartimento Di Oncologia P.O. S. Anna - SS Radioterapia, A.O.U “Città Della Salute E Della Scienza”, Turin, Italy; 12 Radiation Oncology Section, University of Perugia, Perugia, Italy; 13 UO Radioterapia Oncologica, Ospedale S. Chiara, Trento, Italy; 14 Department of Radiation Oncology, IRCCS San Raffaele Scientific Institute, Milan, Italy; 15 Department of Radiotherapy, Ospedale Regionale Parini-AUSL Valle d’Aosta, Aosta, Italy; 16 Department of Translational Medicine, Radiation Oncology Division, University of Pisa, Pisa, Italy; 17 Department of Radiotherapy, Santa Chiara Hospital, Trento, Italy; 18 Radiotherapy Department, Azienda Ospedaliero Universitaria delle Marche, Ancona, Italy; 19 UOC Radioterapia Oncologica, Ospedale Generale Provinciale di Macerata, AST Macerata, Italy; 20 Radiotherapy Unit, Fondazione IRCCS Istituto Nazionale dei Tumori, Milan, Italy; 21 Radiotherapy, A.O.SS. Antonio e Biagio, Alessandria, Italy; 22 Radiotherapy Unit, Ospedale di Circolo Fondazione Macchi, Varese, Italy; 23 Radiotherapy Unit, Ospedale Maggiore di Trieste, Trieste, Italy; 24 Department of Radiation Oncology, General Regional Hospital “F. Miulli”, Acquaviva delle Fonti, Bari, Italy; 25 Radiation Oncology Unit, Department of Oncology, “Santa Maria della Misericordia” University Hospital, Azienda Sanitaria Universitaria Friuli Centrale, Udine, Italy; 26 U.O.C. Radiotherapy, Ospedale Isola Tiberina - Gemelli Isola, Rome, Italy; 27 Department of Radiotherapy, Policlinico Umberto I, Department of Radiological, Oncological and Pathological Sciences, “Sapienza” University of Rome, Rome, Italy; 28 Radiation Oncology Section, University of Perugia and Perugia General Hospital, Perugia, Italy

**Keywords:** Gynecological cancers, Radiotherapy, Acute late toxicity, Prevention, Treatment, Follow-up

## Abstract

Multi-modal therapies for gynecological cancers management may determine a wide range of side effects which depend on therapy-related factors and patient characteristics and comorbidities. Curative or adjuvant pelvic radiotherapy is linked with acute and late toxicity due to irradiation of organs at risk, as small and large bowel, rectum, bladder, pelvic bone, vagina and bone marrow. Successful toxicity management varies with its severity, Radiation Centre practice and experience and skills of radiation oncologists. This position paper was designed by the Italian Association of Radiation and Clinical Oncology Gynecology Study Group to provide radiation oncologists with evidence-based strategies to prevent and manage acute and late toxicities and follow-up recommendations for gynecological cancer patients submitted radiotherapy. Six workgroups of radiation oncologists with over 5 years of experience in gynecologic cancers were setup to investigate radiotherapy-related toxicities. For each topic, PubMed database was searched for relevant English language papers from January 2005 to December 2022. Titles and abstracts of results were checked to verify suitability for the document. Reference lists of selected studies and review papers were added if pertinent. Data on incidence, etiopathogenesis, prevention, treatment and follow-up of acute and late side effects for each organ at risk are presented and discussed.

## Introduction

Today’s multi-modal therapies for gynecological cancers management including surgery, chemotherapy (CHT), external beam radiotherapy (EBRT) and interventional radiotherapy (IR), also called brachytherapy, may determine a wide range of underestimated side effects [1, 2], the development of which depends on therapy-related factors such as radiation therapy (RT) modality and dose, and patient characteristics and comorbidities. Pelvic RT, in the curative or adjuvant setting, is linked with acute and late toxicity due to irradiation of organs at risk (OARs), such as the small and large bowel, rectum, bladder, and femoral heads, and can cause detrimental effects on health and long-term quality of life (QoL) [1, 2]. More recently further toxicities emerged, as hematological, due to the widespread use of concomitant chemoradiation (CRT), and pelvic bone and vaginal side effects [2, 3]. All adverse side effects are scored on specific international scales according to severity of symptoms or clinical evidence, which may vary from minimal to very serious, and even compromise the patient’s survival. The “radiation therapy oncology group” (RTOG) scale [4] and the “common terminology criteria for adverse events” (CTCAE) system [5] were designed to assess acute and late side effects. The subjective, objective, management, analytic/late effects normal tissue task force (SOMA/LENT) scale [6] evaluates only late side effects. QoL questionnaires are often used to subjectively assess patients’ symptoms in relation to their daily life [7, 8]. Successful toxicity management varies with its severity, Radiation Centre practice and the experience and skills of the radiation oncologists which may be limited by a lack of physician education [1].The present position paper was designed by the Italian Association of Radiation and Clinical Oncology Gynecology Study Group **(**AIRO Gyn) to provide radiation oncologists with evidence-based strategies to prevent and manage acute and chronic toxicities and follow-up recommendations for patients with gynecological cancers who underwent RT.

## Methods

With AIRO Steering Committee endorsement, 6 workgroups of radiation oncologists, each including physicians with over 5 years of experience in gynecologic cancer, were setup to investigate early and late RT-related toxicities in the bowel (AB, AP, EG, JDM, AF), rectum (SC, EM, CM, ADA, PF), bladder (FT, RL, GC, AS), bone (EP, CA, MPP, VE), blood (FT, RL, GC, AS), and vagina (MC, VDS, FT, CL) after adjuvant or curative EBRT, with or without BT and/or CHT. The choice of taking part to each group was based on the preference and interest of the single specialists in the specific field of investigation; each group was established during the preparatory meeting. For each topic, PubMed database was searched for relevant English language papers published from January 2005 to December 2023. Search strategy included the following keywords: “cervical cancer*” OR “cervical neoplasm*” OR “cervix cancer*” OR “cervix neoplasm*” OR “uterine cancer*” OR “uterine neoplasm*” OR “vaginal cancer*” OR “vaginal neoplasm*” OR “vulva* cancer*” OR “vulva* neoplasm*” OR “endometrial cancer*” OR “endometrial neoplasm*” OR “ovarian cancer*” OR “ovarian neoplasm*” OR “Genital Neoplasms, Female” [Mesh]. An example of search strategy referring to bone toxicity is shown in Table 1. Titles and abstracts of literature search results were checked to verify suitability for the document. Reference lists of selected studies and review papers were manually searched for additional pertinent publications. Editorial, abstract from international meetings and case reports/series were excluded. Results were grouped according to the topic investigated. Data on incidence, etiopathogenesis, prevention, treatment and follow-up of acute and late side effects for each OAR are presented and discussed.

**Table 1:** Example of search strategy referring to bone toxicity

Keywords	
“radiotherapy technique*” OR “radiotherapy timing” OR “positioning device*” OR “pharmacological intervention*” OR “non-pharmacological intervention*” OR “Radiation Injuries/prevention and control”[Mesh] AND “radiation toxicity*” OR “radiation toxic effect*” OR “complication*” OR “adverse effect*” OR “pelvic bone” OR “osteonecrosis” OR “radionecrosis” OR “pain” OR “fracture*” OR “Pelvic Bones/radiation effects”[Mesh] AND “cervical cancer*” OR “cervical neoplasm*” OR “cervix cancer*” OR “cervix neoplasm*” OR “uterine cancer*” OR “uterine neoplasm*” OR “vaginal cancer*” OR “vaginal neoplasm*” OR “vulva* cancer*” OR “vulva* neoplasm*” OR “endometrial cancer*” OR “endometrial neoplasm*” OR “ovarian cancer*” OR “ovarian neoplasm*” OR "Genital Neoplasms, Female”[Mesh]	

## Results

### Bowel toxicity

#### Incidence and etiopathogenesis

**Fig. 1:**
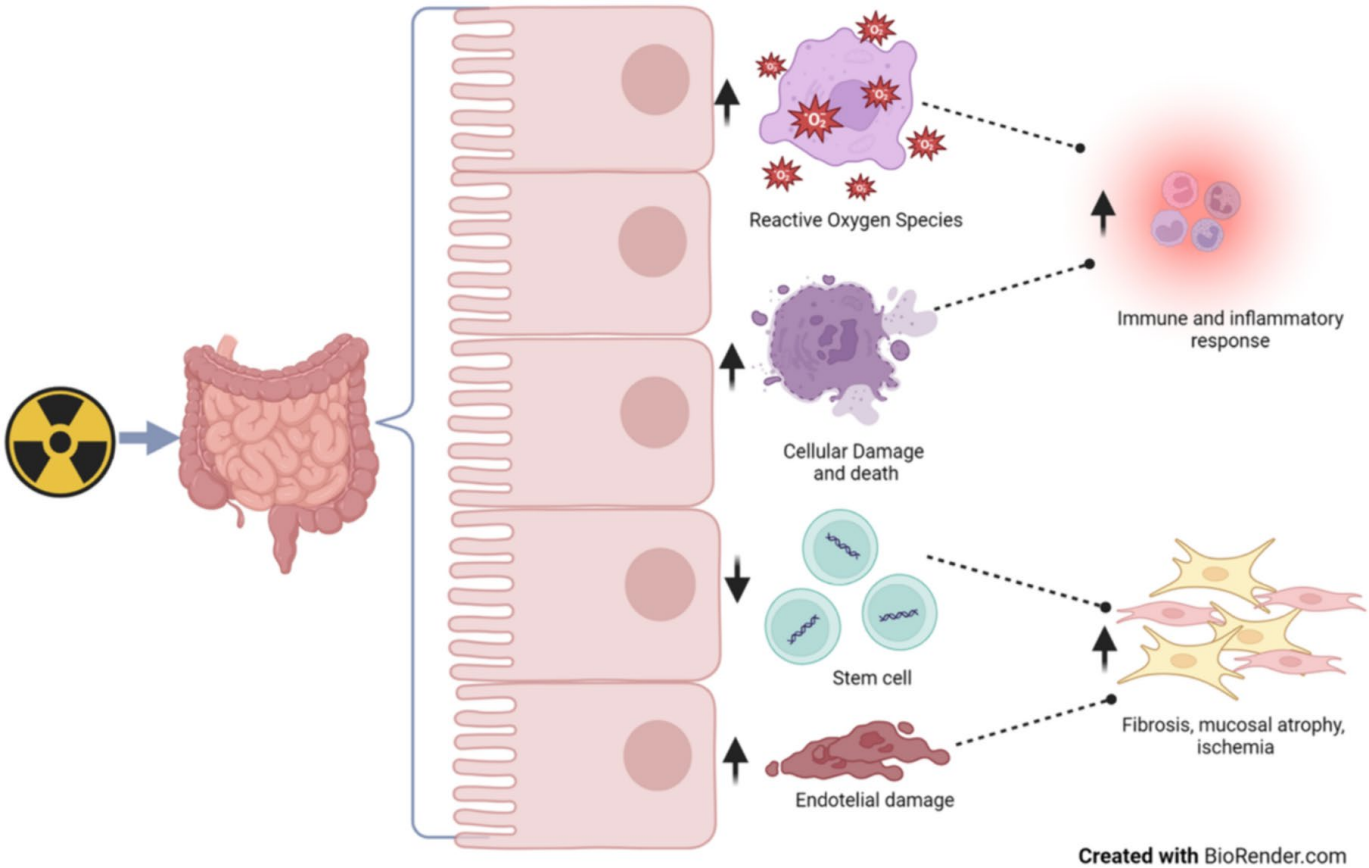
Etiopathogenesis of bowel toxicity

Overall, small bowel toxicity develops in up to 55% of women during RT or within 3 months of it and in 15% after more than 3 months [9, 10], limiting dose delivery and negatively impacting QoL [11, 12]**.** Although the etiopathogenesis of enteritis after abdominal RT is still unknown, changes in fecal microbiota have recently been hypothesized to be involved [13]. RT induces cellular damage, cell death, and generation of reactive oxygen species, thus triggering secondary reactive inflammatory processes and immune responses. Moreover, stem cell depletion and microvascular alterations induce progressive tissue fibrosis, ischemia, and mucosal atrophy [9]. Occurrence of enterocolitis and diarrhea were reported at the end of treatment in 51.9% of endometrial and cervical cancer patients treated with 3D conformal RT (3D-CRT) and 33.7% of patients treated with IMRT [14]. No certain data are available on the real incidence of bowel toxicity on vulvar and vaginal cancers due to their rarity. Bowel toxicity was not reported in a large multi-institutional series of vulvar cancer patients who had received adjuvant RT with or without CHT [15]. A few cases of acute and late toxicity, not exceeding G3, were observed in other series of adjuvant, preoperative or definitive RT in vulvar cancer patients. Only G4 skin toxicity was found [16–18]. Usually occurring after 2 weeks of RT, diarrhea was related to dose per fraction and irradiated volume. Although it may be underestimated, chronic RT-related enteritis was reported in up to 20% of patients [19], generally from 18 months to 6 years after treatment. Most symptoms were due to alterations of the bowel vascular compartment leading to the most serious side effects, i.e., ischemia, progressive intestinal fibrosis, stenosis and/or fistulas. Etiopathogenesis of bowel toxicity is shown in Fig. 1.

#### Prevention

Pharmacological and RT techniques may prevent small bowel toxicity. Several studies demonstrated that probiotics during treatment significantly reduced acute toxicity [11]. A double-blinded study of 54 patients who underwent pelvic RT assessed probiotics against placebo [12]. During EBRT and in the three weeks afterward, episodes of diarrhea and abdominal pain were evaluated through interviews and questionnaires and scored on the CTCAE scale [5]. Probiotics significantly reduced not only the incidence of diarrhea more than placebo (53.8 *vs* 82.1%, *p* < 0.05), but also its severity (*p* < 0.05) and the need for loperamide administration (*p* < 0.01) [12]. Furthermore, probiotics were associated with a significant difference (*p* < 0.001) in grade 2 abdominal pain and in the number of daily episodes of abdominal pain [12]. Other studies [20, 21] reported similar results, linking probiotics with a significant difference in use of loperamide (32% *vs* 9%) [21]. Nutritional supplements based on Zinc, Prebiotics, Probiotics and Vitamins [22]**,** amifostine [23] and the oral CXCR4 Inhibitor X4-136 were also reported to be useful in patients treated with pelvic RT not only for cervical and endometrial cancer but also for anal and rectal cancer [24].

Small bowel toxicity is reduced by modern RT techniques such as IMRT, volumetric modulated arc therapy (VMAT), tomotherapy and proton beam RT [16, 17, 25–31]. On the other hand, changes in setup positions (supine *vs* prone) yielded discordant results [32–35].

Hoover et al. found that visceral adipose-corrected bowel bag dosimetric constraints correlated better with acute bowel toxicity than the current standard practice of considering V45 cc and V40% [36]. Using image-guided radiotherapy (IGRT), Xin et al. [37] evaluated setup errors and their effects on acute bowel toxicity and treatment efficacy in 170 cervical cancer patients who underwent IMRT ± IGRT. Response rates were similar in both groups, but IGRT significantly corrected and reduced setup errors during treatment and enhanced the accuracy of dosage distribution within OARs (such as targeted regions), thus reducing RT-related toxicity [33]. Park et al. [38] found bladder filling associated with the use of personalized immobilization devices and the adoption of the prone position in 3D-CRT displaced the small bowel continuously away from the irradiated field in cervical cancer patients. Adopting these precautions decreases the amount of intestine exposed to radiation and consequently can decrease the frequency and severity of onset of side effects [38].

#### Treatment

Small bowel toxicity may have an impact on treatment compliance, requiring symptomatic therapy when necessary. Treatment of acute small bowel toxicity can require probiotics to restore intestinal microbiota, loperamide and dietary counseling, bearing in mind that nutrient malabsorption may occur as a late side effect [39].

#### Follow-up

During follow-up, all patients should be evaluated to assess late toxicity for early intervention by a specialist multidisciplinary team (e.g., gastroenterologist, nutritionist, surgeon). Patients recovering from initial complications remain at risk of late and persistent adverse events [40].

Summary of evidences is shown in Table 2.

**Table 2:** Summary of evidence on specific toxicity

Toxicity/	Etiopathogenesis	Symptoms	Incidence	Treatment	RT prevention	Follow-up
Intestinal	Cellular damage and death, reactive inflammatory processes, stem cell depletion, microvascular alterations, progressive tissue fibrosis, ischemia, and mucosal atrophy [9] Changes fecal microbiota [13]	Enterocolitis diarrhea [14] Malabsorption, ischemia, progressive intestinal fibrosis, stenosis and/or fistulas [15]	Acute 37–55% [9, 10] Late up to 20% [15]	Probiotics to restore microbiota [11, 12, 16, 17] Dietary recommendations Loperamide administration [35]	IMRT, VMAT, tomotherapy, proton beam RT, IGRT [21–27, 33] Adipose-corrected bowel bag dosimetric constraints [32] Bladder filling and personalized immobilization devices, adoption of the prone position in 3D-CRT [34]	Late toxicity evaluation by a multidisciplinary team Risk of late toxicity if occurrence of early toxicity [36]
Rectal	Inflammatory process of the superficial mucosa [39], loss or distortion of the microvillus architecture with hyperemia, edema, and ulceration, arteriole endarteritis, full-thickness ischemia, submucosal connective tissue fibrosis, neoangiogenesis [38, 40]	Diarrhea, cramps, tenesmus, urgency, mucus discharge, and minor bleeding [39] Bleeding, tenesmus, defecation difficulties, strictures, perforation, fistula, rectal obstruction [37, 41]	Acute up to 20% [43] Late 5–20% [37] Acute up to 20%	Topical anti-inflammatory drugs alone or combined with steroids [60] Hyperbaric oxygen if soft tissue necrosis or chronic proctitis [61–64] Potassium titanyl phosphate, Argon and YAG lasers [65, 66]. Radio-Frequency Ablation Cryoablation for hemostasis [67, 68]	IMRT [44–47] IGRT [48, 52, 53] NO differences in pts position (supine vs prone) [29] CTV-PTV margin shrinkage [49–51]	Sigmoidoscopy if bleeding or evidence of occult fecal blood [69, 70]
Urinary	Damage to bladder vasculature and smooth muscle fibers, resulting in edema, cell death and fibrosis [2, 3]	Dysuria, urinary frequency, nocturia, hesitancy infection, discomfort, hematuria, incontinence [2, 3, 71–73]	50% [2, 3, 71]	Hydration, non-steroidal anti-inflammatory drugs, anticholinergic agents [2] If drug therapy is uneffective: -Botulinum toxin A injection into the detrusor muscle [2] - Hyperbaric oxygen, clot evacuation, endoscopic fulguration and bladder irrigation [80] -Surgery (percutaneous nephrostomy or ureteral stent or ileal ureteral substitution [80])	IMRT, etc. IGRT Ureteral dose of D0.1 cc < 23.1 Gy EQD2 [76]. Bladder D2cm3 ≤ 80 Gy EQD2 [79]	Clinical examination and accurate anamnesis for guiding further instrumental tests for urinary tract dysfunction
Bone	Osteoblast death, increased osteoclast activity, trabecular bone loss [87, 88], reduced BMD, osteoporosis, fractures [1, 86, 89–94]	Bone demineralization, osteoporosis [1, 86, 89–94] Pain due to fractures [93–96]	Largely underestimated [86] 3–37% [91, 92, 97–99]	Diagnostic MRI Analgesic drugs Bed rest [2, 95]	Pre-RT BMD assessment and correction [85, 109] Bone sparing IMRT [92, 96, 98, 106, 111] Limit maximum doses [92] Sacrum D50% < = 35 Gy, EBRT 45 Gy tighter margins [91]	Consider patient’s reported symptoms [110] BMD assessment Bone loss correction [89, 93, 112, 148] MRI when necessary [113, 114]
Hematological	RT causes damage to almost all hematopoietic stem cells, as well as reducing the hematopoietic capacity of hematopoietic progenitor cells, which can accelerate the incidence of hematotoxic events [118–123, 125] Association of RT and a myelosuppressive CHT [117–121]	Leukopenia (in particular lymphopenia) Anemia Thrombocytopenia [118–123, 125]	> G2: 30–45% [145]	Biochemistry investigations Administration of growth factors Blood transfusions [119, 121, 147]	Pelvic bone marrow sparing RT techniques [141] Dosimetric parameters to reduce hematological toxicity: V10 < 75–95%, V20 < 65–80%, V40 < 28–37% [146]	Routine analysis should include routine blood and biochemistry tests other than CT Scan, USG abdomen, ECG and chest X-ray [145, 147]
Vaginal	Microcirculatory alterations, atrophy, telangiectasia adhesions, fibrosis [153]	Vaginal bleeding, vaginal dryness [151], vaginal stenosis [153, 166] dyspareunia, pain during sexual practice, urogenital symptoms [152]	Vaginal atrophy 50–60% [151] 22% actuarial probability of vaginal stenosis at 2 years [153]	Topical application of hyaluronic acid, along with vitamin E and A [9, 162–165] Hormone replacement therapy [9, 160, 168, 169] Pelvic floor muscle exercises [174] Ovarian preservation [160, 161]	Using 3D BT volumetric planning [155] De-escalating the dose to the ICRU rectovaginal point from 75 to 65 Gy [157] Doses < 50 Gy to the posterior inferior border of the pubic symphysis with EBRT + BT [158]	Consider Patient Reported Outcome for vaginal and sexual symptoms [154]

BMD = Bone Mineral Density, BT = Brachytherapy, CHT = Chemotherapy, 3D-CRT = 3D-Conformal Radiotherapy, EBRT = External-Beam Radiation Therapy, IGRT = Image-Guided Radiation Therapy, ICRU = International Commission on Radiation Units and measurements, IMRT = Intensity Modulated Radiation Therapy, MRI = Magnetic Resonance Imaging, RT = Radiotherapy, VMAT = Volumetric Modulated Arc Therapy

### Rectal toxicity

#### Incidence and etiopathogenesis

**Fig. 2:**
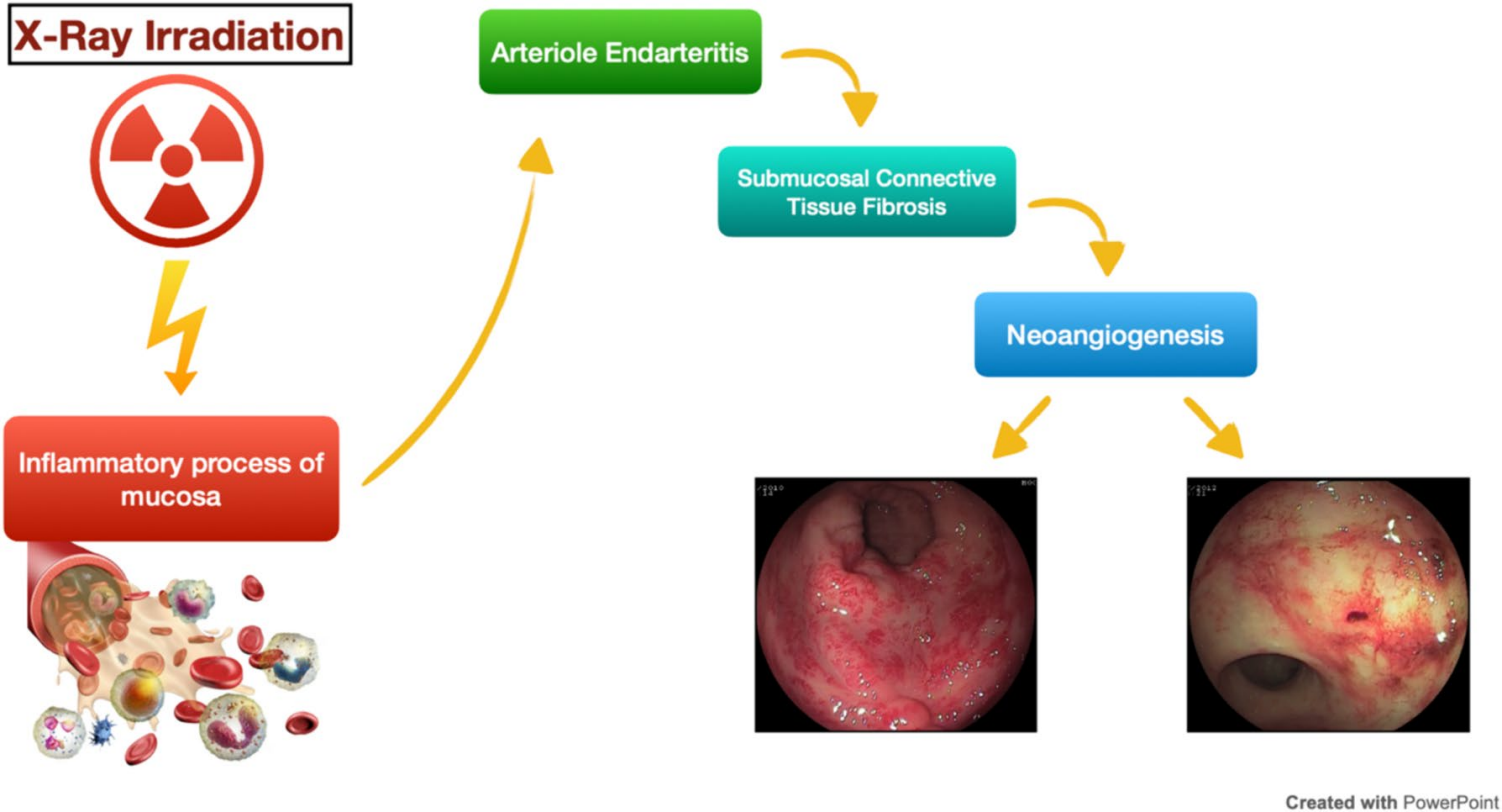
Etiopathogenesis of rectal toxicity (Leukocytes images from: mypersonaltrainer.it; Colonoscopy Images from: https://www.medicitalia.it/minforma/gastroenterologia-e-endoscopia-digestiva/1923-proctite-attinica-terapiaendoscopica-con-argon- plasma-coagulation.html)

RT-related proctitis, a common complication of pelvic RT, is due to the rectal proximity to pelvic organs and its fixed position [41]. Although the incidence is not clear, due to a lack of consensus on its definition and reporting methodologies, large irradiated volume, RT dose (< 45 Gy or above 70 Gy), older RT technique (3D-CRT vs IMRT), are generally agreed to be risk factors [42]. Acute RT-related proctitis occurs almost immediately after starting RT and lasts for up to 3 months. It is an inflammatory process affecting the superficial mucosa and its symptoms usually include diarrhea, cramps, tenesmus, urgency, mucus discharge, and minor bleeding which typically resolve spontaneously following completion of treatment [43]. Even though chronic RT-related proctitis may begin during the acute phase of radiation proctitis, symptoms may not become apparent until a median of 8–12 months after completing RT [42]. It is histologically characterized by arteriole endarteritis, submucosal connective tissue fibrosis and neoangiogenesis followed by telangiectasias [44]. Bleeding is the most common symptom; strictures, perforation, fistula and rectal obstruction may also occur [41, 45]. In some cases, loss of distensibility, due to rectal wall fibrosis, results in tenesmus or defecation difficulties. Etiopathogenesis of rectal toxicity is shown in Fig. 2.

#### Prevention

Rectal toxicity should be prevented because it may interrupt treatment, limit the delivered RT dose with a consequent reduction in treatment efficacy and worsen the patient’s QoL [46]. Prevention should begin by assessing the individual patient’s risk profile bearing in mind that comorbidities, such as diabetes mellitus, vascular disease, arterial hypertension, atherosclerosis, inflammatory bowel disease, collagen disease, and HIV infection, are associated with increased risk of toxicity [41].

RT-related rectal toxicity is reduced by decreasing the dose delivered to the rectum and by adopting strategies that modulate cellular and tissue responses to RT, thus reducing radiosensitivity [14, 47].

Several trials demonstrated that IMRT was associated with less rectal toxicity than 3D-CRT [48–50]. A prospective, phase III trial was conducted on 234 patients with cervical or endometrial cancer who were randomized to post-operative RT with IMRT or 3D-CRT. IMRT was associated with significantly fewer episodes of diarrhea and fecal incontinence [51]. The Post-operative Adjuvant Radiation in Cervical Cancer (PARCER) phase III randomized trial, which compared late toxicity in women with cervical cancer undergoing post-operative RT with IGRT-IMRT or 3D-CRT, demonstrated that IGRT-IMRT significantly reduced late toxicity with no difference in disease outcomes [52].

Although clinical target volume-planning target volume (CTV-PTV) margin shrinkage might reduce RT-related toxicity, too narrow margins could increase the risk of geographic miss, especially with IMRT/VMAT techniques with highly conformal doses to the target volume [53–55]. IGRT reduces the risks of target miss and/or OARs overdose during RT delivery [56]. The role of cone-beam computed tomography (CT) [57] was evaluated in 170 patients with cervical cancer to check setup errors and their effects on acute toxicity and RT efficacy. The results showed it corrected and reduced setup errors, improved dose distribution accuracy in the target area and OARs, significantly reduced toxicity and improved efficacy [57]. Even though prone and supine positions were not associated with any differences in dosimetry and rectal toxicity with IMRT, the supine position is preferred because of fewer setup uncertainties and greater patient stability during treatment [33].

Several drugs have been used to prevent RT-related toxicity by modulating the radiosensitivity of normal tissues [47]. Administered intravenously, subcutaneously or intrarectally (the most effective route) [44], amifostine exerts radioprotective efficacy through diverse complex and not fully understood molecular and cellular processes, which are hypothesized to include free-radical scavenging, DNA protection, DNA repair acceleration, and induction of cellular hypoxia [58]. It may up-regulate the expression of proteins that repair DNA and inhibit apoptosis through Bcl-2 and hypoxia-inducible factor-1α [59]. Several small, single-center controlled trials suggested that amifostine may reduce acute gastrointestinal toxicity during pelvic RT, while there does not appear to be any meaning reduction in late morbidity. Thus, despite many studies [14, 60, 61] which a recent review judged to be at high risk of bias [62], due to methodological limitations and very uncertain evidence, amifostine has not been associated with sufficiently reduced side effects to satisfy FDA regulatory requirements [59].

The present position concurs with the MASCC panel’s recommendation that cytoprotective agents like Sucralfate, non-steroid anti-inflammatory agents like balsalazide, mesalazine and prostaglandin analog like misoprostol should not be treatment of choice to prevent radiation-induced proctitis, due to conflicting evidence on their efficacy [63].

#### Treatment

Grade 1/2 proctitis responds to topical anti-inflammatory products, such as sulfasalazine or mesalazine alone or combined with steroids [64].

Hyperbaric oxygen which induces neo-vascularization, tissue re-oxygenation, collagen neo-deposition and fibroblast proliferation, elicited responses in the majority of patients with soft tissue necrosis or chronic proctitis [65–67]. A review evidenced that hyperbaric oxygen therapy may improve outcomes, but further studies are necessary to establish the correct patient’s selection [68]. Potassium titanyl phosphate, Argon and YAG lasers are used to treat superficial injuries [69]. Repeated applications of Argon Plasma Coagulation resolved 80–90% of cases with chronic proctitis and bleeding [69, 70]. Anal or rectal pain in 20% of cases resolved spontaneously while sever complications like hemorrhage, necrosis and perforation occurred in 10% of cases [69]. Two or 3 sessions of Radio-Frequency Ablation provided hemostasis without severe complications [71]. Cryoablation yielded excellent results but is not in widespread use [72]. Refractory proctitis requires surgery leading to colostomy or exenteration.

#### Follow-up

Sigmoidoscopy is recommended for investigating patient-reported bleeding or evidence of occult fecal blood [73, 74].

Summary of evidences is shown in Table 2.

### Urinary toxicity

#### Incidence and etiopathogenesis

After pelvic RT for gynecologic malignancies about 50% of women experience acute urinary symptoms, including dysuria, urinary frequency, nocturia, and hesitancy which are linked to RT-induced cystitis. Urinary disturbances occur after a dose of 20 Gy to the bladder and subside 2–3 weeks after the end of treatment [2, 3].

The bladder and urethra frequently show signs of late radiation damage, leading to urinary sequelae like infection, discomfort, and hematuria. Reduced bladder capacity leading to frequent urination is due to damage to bladder vasculature and smooth muscle fibers, resulting in edema, cell death and fibrosis [2, 3].

Bladder dysfunction occurring many years after RT, affects the patient's QoL and includes urgency, frequency and incontinence due to high dose bladder neck irradiation (26%), ureteral stricture or fibrosis (1–3%), hemorrhagic cystitis (5–9%), but rarely vesicovaginal and ureterovaginal fistulas [2, 3, 75]. Chronic symptoms appear to be the result of vascular endothelial cell damage that develops with a latency period of 1 to 25 years.

The risk of late genitourinary toxicity increased with a history of abdominal surgery, pelvic inflammatory disease, hypertension, diabetes mellitus and smoking [76]. Older age significantly impacted incontinence, because shorter vaginal lengths can result in higher bladder neck doses. Obesity and overweight were risk factors for incontinence and frequency [77].

Most RT-related ureteral strictures caused by RT affect the distal portion of the ureter, and it was demonstrated that delaying the clearance of ureteral blockage increases the risk of serious long-term morbidity, including infections, kidney damage, and arterial hypertension. The risk of ureteral stricture in patients with locally advanced cervical cancer and hydronephrosis at diagnosis was 11.5% at 5 years compared 4.8% without hydronephrosis [78]. A higher incidence of ureteral stricture was seen in patients who underwent hysterectomy or other pelvic surgeries followed by RT. In the EMBRACE investigations, however, despite 26.7% of patients having received laparoscopic staging [78], a link between surgery and ureteral stricture was not observed, after EBRT with or without node boost and Image-Guided Adaptive IR.

**Fig. 3:**
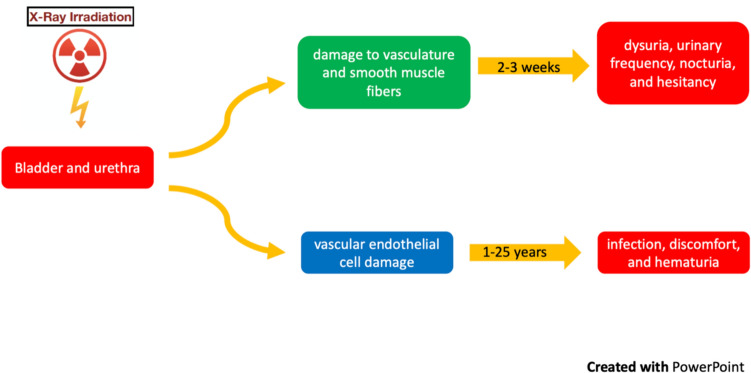
Etiopathogenesis of urinary toxicity

Diverse urinary morbidity endpoints exhibit different temporal trends, as shown by the EMBRACE research [78]. This suggests that a wide range of intricate physiological mechanisms develop during radiation. The exposure of various organ sub-volumes to RT, the differences in dose–effect relationships for various symptoms, the potential reversibility of some late effects, and the effective management of late effects are additional factors that influence the development of treatment-related morbidity. Etiopathogenesis of urinary toxicity is shown in Fig. 3.

#### Prevention

Different IMRT modalities may reduce the rate of acute and late high-grade toxicity [79, 80]. On the other hand, Dröge et al. reported that patients treated with VMAT experienced acute < grade 3 urinary toxicity more frequently compared with 3D-CRT, probably due to the larger amount of irradiated bladder wall [81].

In patients with locally advanced cervical cancer, who were treated with EBRT, CHT and IR the investigators of EMBRACE Collaborative Group found ICRU bladder point (ICRU-BP) dose > 75 Gy was a stronger predictor of incontinence than bladder D2 cm^3^ since it is located near the trigone, bladder neck and urethra [77]. A ureteral dose of D0.1 cc of 23.1 Gy EQD2 is connected to a 10% chance of G3 or greater urinary toxicity [82]. To reduce the incidence of severe urinary complications to at least 15%, a D2cm^3^ ≤ 80 Gy EQD2 should be used. Dose to the bladder trigone was also predictive of severe late urinary toxicity [83].

#### Treatment

Guidelines for managing urinary toxicity are lacking. For acute symptoms, the workup should include urine analysis and urine culture. Low-grade urinary symptoms are managed with non-steroidal anti-inflammatory drugs, anticholinergic agents such as oxybutynin, or analgesics such as phenazopyridine. Botulinum toxin A injection into the detrusor muscle may be used when drug therapy is ineffective [2]. Symptoms are generally self-limited, and drugs can be discontinued as symptoms improve. Treatment for hemorrhagic cystitis includes hydration, hyperbaric oxygen, clot evacuation, endoscopic fulguration and bladder irrigation with a variety of substances [84]. Surgery should be evaluated in case of refractory disease. Infection and primary bladder malignancy must also be evaluated.

Ureteral strictures, if not due to recurrent disease, are repaired with endoscopy or open surgery including percutaneous nephrostomy or ureteral stent or ileal ureteral substitution [84] which can be challenging due to the poor vascularity and wound healing following radiation. Vesicovaginal fistulae, not related to disease, may require fulguration and drainage or surgery [84].

#### Follow-up

In addition to the clinical examination, the accurate anamnesis guides the specialist in any ulterior investigation with further instrumental tests for urinary tract dysfunction. Bladder cystitis and bleeding may reach a peak prevalence rate at about 30 months, after which prevalence rates fell to baseline, indicating healing [85].

Summary of evidences is shown in Table 2.

### Bone toxicity

#### Incidence and etiopathogenesis

Surgery with ovary removal, CHT and RT may have detrimental effects on bone mineral density (BMD) leading to osteoporosis and fractures which impact on quality of life and life expectancy [86–89]. The incidence of bone toxicity after RT or CRT is largely underestimated because attention has only recently focused on long-term cancer survivors [90].

RT is hypothesized to be linked to osteoblast death and less activity as well as increased osteoclast activity and inflammatory cytokine release. Consequences include bone marrow adiposity, trabecular bone loss [91, 92], reduced BMD, osteoporosis, and pelvic insufficiency fractures (PIF) [1, 90, 93–98].

The incidence of PIF after RT ranges from 10 to 14% [97–100], but other studies reported incidences ranging from 3% to 37.4% [95, 96, 101–103], with a higher incidence in patients over 50 years old [95]. Median time to PIF occurrence ranges from 7 to 39 months [97, 99, 101, 104]; actuarial rates increase from 3.6% at 1 year to 15.7% at 3 years [93].

**Fig. 4:**
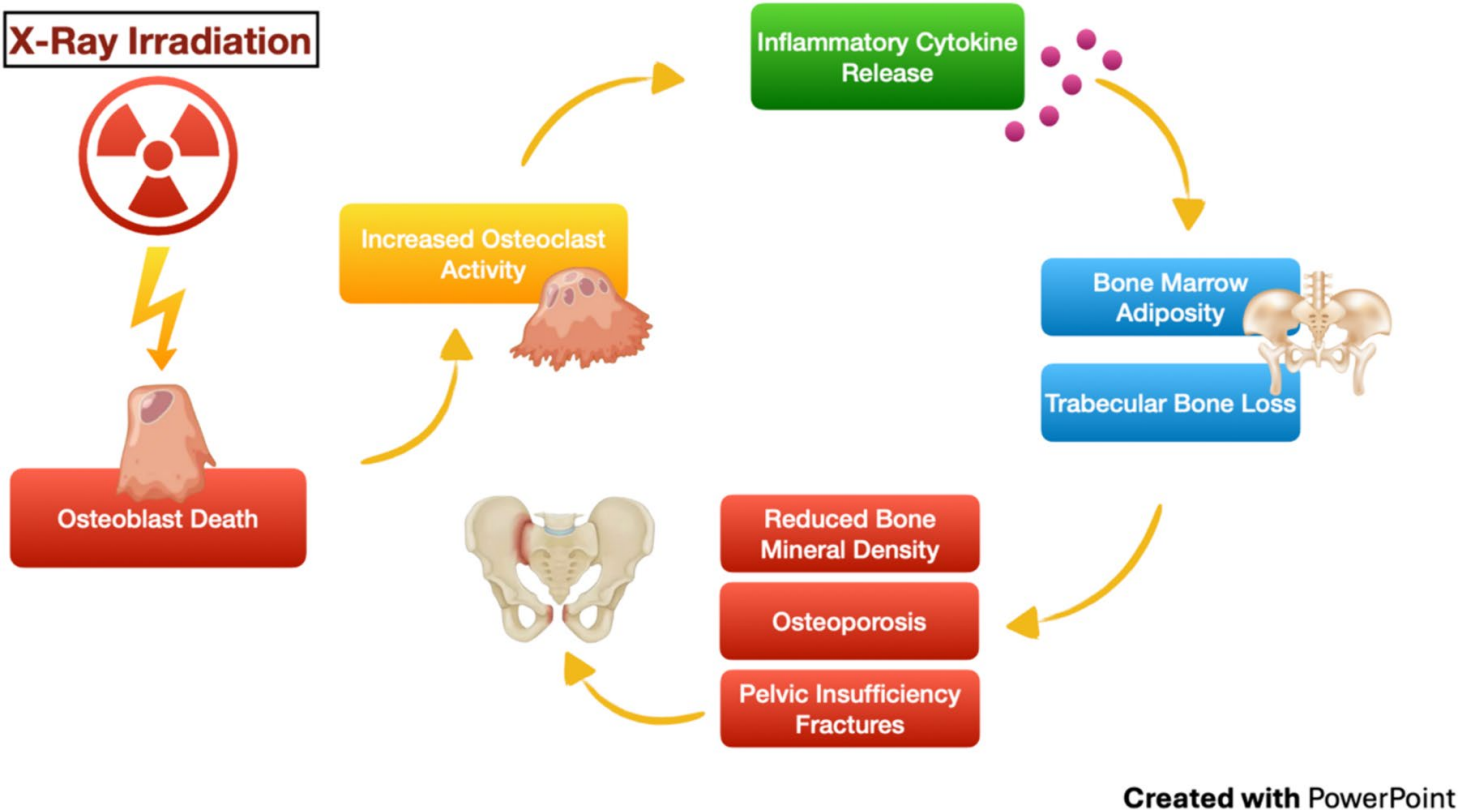
Etiopathogenesis of bone toxicity (Images from: https://depositphotos.com/it/vectors/osteoblasti.html; https://www.fisioterapiaitalia.com/patologie/bacino/fratture-del-bacino)

PIF is diagnosed on evidence from X-rays, bone scans, CT scans, or magnetic resonance imaging (MRI), with MRI being the most reliable tool [92, 93, 95, 103]. The sacrum, sacroiliac joint and pubis are the most frequently affected sites [97, 99, 101]; more than 1 PIF can occur [95]. About 50–70% of patients with PIF refer pain [95, 96, 99, 102]. Risk factors for PIF development are age over 50 [93, 98, 101, 105], post-menopause [96, 97, 106], low BMD at baseline and after RT [86, 99, 100, 104, 107], low body weight/low body mass index [86, 101, 102, 108], osteoporosis [93, 108, 109], high alkaline phosphatase level at baseline [93]. RT-related parameters include treatment modality (IMRT *vs* 3D-CRT), and intent (curative or adjuvant) which correlate with the delivered doses [96, 100, 102, 110]. Etiopathogenesis of bone toxicity is shown in Fig. 4.

#### Prevention

Before RT, primary prevention of PIF is based on accurate evaluations of BMD and risk factors, particularly in postmenopausal women and in patients over 50 years old [89], as lower pre-treatment CT bone density was found in patients developing PIF [104, 105, 107] and a global reduction of BMD was reported after RT or CRT, even though there is no consensus on whether adding CHT to RT increases the risk of PIF [86, 95, 102, 109–112]. When necessary, therapy should be prescribed, e.g., vitamin D, calcium, bisphosphonate and, in selected cases, hormone replacement therapy [89, 113].

RT-related bone toxicity should be minimized even though to date modalities and doses have not yet been clearly defined and no dosimetric constraints are available for the pelvic bone dose to reduce the incidence of bone toxicity and PIF [114]. In cervical cancer patients treated with curative intent, IMRT plus IR was associated with less PIF than 3D/CRT plus IR [100, 102, 110, 115]. This difference did not emerge in the adjuvant setting [109], due to the lower doses administered in the post-operative treatment.

Controversial results were achieved when a simultaneous integrated boost (SIB) was administered by IMRT [96, 103]. Bazire et al. [96] found maximum doses were significantly higher at fracture sites than in pelvic bones without PIF; while, Mir et al. [103] reported 60 Gy SIB did not impact fracture sites. Ramlov et al. [95] found sacrum D50% was a significant risk factor for sacral fracture in patients over 50 years old who underwent curative RT for locally advanced cervical cancer, indicating that high doses to the total bone and not just to a small part can cause PIF. Indeed, reducing sacrum D50% from 40 to 35 Gy lowered the risk of sacral PIF from 45 to 22%. Finally, to prevent PIF the recommended EBRT dose should be reduced to 45 Gy [95] and tighter margins should be applied when contouring. An internal margin of 3 mm for pelvic bone, called bone − 3 mm, was used to assure that PTV did not extend beyond it by Bazire et al. who reported a PIF incidence of 3% and 4% for cervical and endometrial cancers, respectively, using IMRT [96]. A nomogram was proposed to predict the risk of sacral PIF based on age and V40G_3_ (EQD2 *α/β* = 3), which were found predictive factors for PIF in patients receiving adjuvant or radical RT [103].

#### Treatment

Management of bone toxicities and PIF requires a multidisciplinary approach. Preventive therapy for low BMD and osteoporosis should continue throughout treatment and follow-up [2]. PIF is generally treated with no steroidal anti-inflammatory drugs, analgesics or opioids, if necessary; treatment can take many months [99]. Bed rest is indicated to avoid load with slow full mobilization [2]. Hospitalization is required for about 10% of cases [99] and femoral head fractures require surgery [2, 99]. Specific bone therapies improve PIF repair [99] and physiotherapy may be required [2].

#### Follow-up

Follow-up examinations should include regular BMD assessment and drug therapy for patients at risk [93, 97, 112, 116]. Attention should be paid to patient-reported musculoskeletal symptoms, which are often overlooked as specific QoL questionnaires do not investigate RT-related bone toxicity [114]. Imaging studies, particularly MRI, should be prescribed for symptomatic patients, taking care to differentiate PIF from metastases [117, 118].

Summary of evidences is shown in Table 2.

### Hematological toxicity

#### Incidence and etiopathogenesis

Due to the heterogeneity of gynecological cancers and the range of treatments (EBRT alone, IR alone, or combined, with or without CHT), no studies have defined the impact of each factor on the incidence of hematological toxicity. Several studies reported that bone marrow (BM) acted as a parallel organ and emphasized the need for sparing a threshold of its volume. Predictors contributing to hematological toxicity were: baseline white blood cells, absolute neutrophil count, hemoglobin and platelets; use of para-aortic irradiation; body mass index. No associations were found between hematological toxicity and race, age, comorbidity, performance status, smoking history, stage, BM volume, pre-treatment transfusions [119, 120]. Hematological toxicity might depend on the association of RT and a myelosuppressive CHT regimen [121]. In the setting of CRT for various pelvic cancers, including cervical cancer [122–125], myelosuppressive CHT was identified as the primary cause of anemia, leukopenia, and neutropenia [122–125] which, together with thrombocytopenia, are common and, at times, life-threatening side effects of oncologic treatments for pelvic malignancies [122–127]. Huang et al. showed hematological toxicity grade 2 or higher in 69.5% of cervical cancer patients undergoing CRT with standard RT; while, hematological toxicity grade 2 or higher was 50% lower in patients undergoing BM sparing with IMRT [128]. Hematological toxicity is also caused by incidental BM irradiation during pelvic nodal RT due to radiosensitivity of BM stem cells [122–127], with leukopenia, and in particular lymphopenia, being major consequences [129]. BM composition (particularly the fat fraction) was reported to change during RT [130, 131], with the decline and regeneration of active, red BM (aBM) being RT dose-dependent [124]. Patients with a low pre-treatment aBM volume, identified by 18F-FDG-PET-CT and the technetium-99 m (Tc-99 m) sulfur colloid SPET, were more likely to develop hematological toxicity grade 3 than patients with a larger aBM volume before irradiation [132, 133].

aBM, half of which is located within pelvic bones and lumbar vertebrae [122, 124, 125], is highly radiosensitive as just 4 Gy reduces its volume by 50% within 1 or 2 weeks [134, 135]. Indeed, a dose threshold of 4 Gy, with no benefit from fractionation, was reported for BM suppression in pelvic cancer patients undergoing CRT with IMRT [134]. Continuous lymphoid hematopoiesis within aBM [129, 136], is especially vulnerable to RT [135, 137]. The lethal radiation dose that reduces the surviving lymphocyte fraction by 50% (LD50) is just 1.5 Gy, and the LD90 is just 3 Gy [138].

**Fig. 5:**
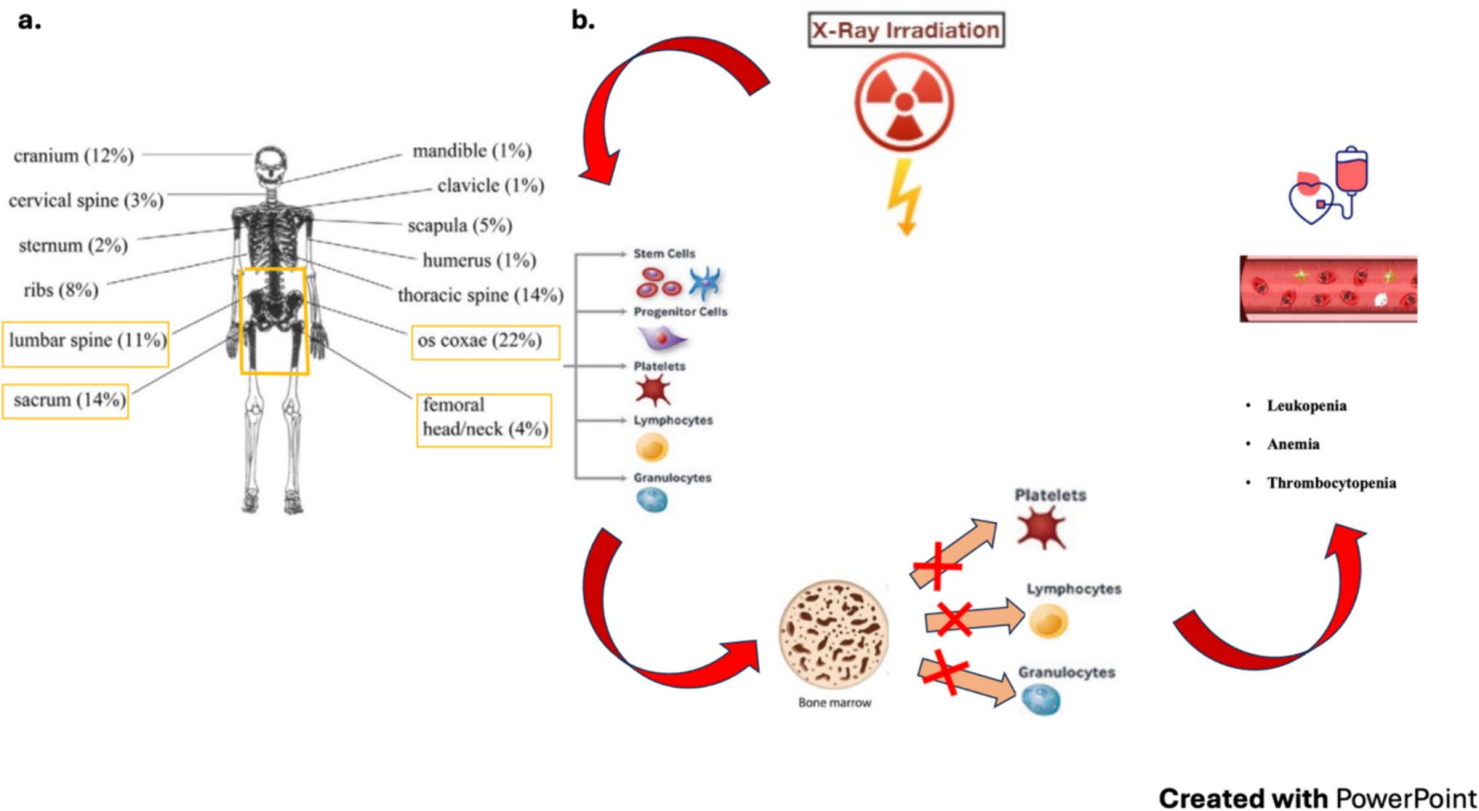
**a** Distribution of bone marrow in an adult; more than one- half of the body’s bone marrow (BM) is located in the os coxae, sacrum, proximal femora, and lower lumbar spine (these areas are included in the treatment volume with pelvic RT) (Images from: https://doi.org/10.1016/j.ijrobp.2006.03.018). **b** Etiopathogenesis of hematological toxicity

Even though avoiding BM during RT appears to be a factor in preserving aBM and decreasing hematological toxicity [139], BM tolerance remains poorly understood [121]. Moreover, BM was excluded from normal tissue dose constraint guidelines such as “the Emami table” [140] or Quantitative Analysis of Normal Tissue Effects in the Clinic (QUANTEC) [141]. Furthermore, the Lyman–Kutcher–Burman model, the most widely used normal tissue complication probability (NTCP) model, does not consider BM toxicity [142]. Etiopathogenesis of hematological toxicity is shown in Fig. 5.

#### Prevention

Currently, the development of effective pelvic BM sparing RT techniques is limited due to a lack of knowledge on the spatial location of BM to be saved and the required degree of sparing that is essential [143]. In the future proton therapy may be beneficial to enable BM sparing due to its physical characteristics and ability to achieve satisfactory target dose distribution [144].

A systematic review investigating the clinical benefit of aBM sparing in cervical cancer patients receiving CRT evidenced decreasing incidence of hematological toxicity [145]. Since functional imaging to identify aBM by 18F-FDG-PET-CT and the technetium-99 m (Tc-99 m) sulfur colloid SPET is expensive and not commonly available, earlier studies proposed an atlas-based method for delineating the aBM in patients with cervical cancer for BM sparing IMRT [146, 147]. Different methods were proposed for delineating pelvic bones: delineating the external contour of all bones within the pelvis or utilizing specified CT window settings or anatomical landmarks [124]. Several studies recommended the following dosimetric parameters for pelvic bones to reduce hematological toxicity: V10 < 75–95% [125, 148], V20 < 65–80% [148, 149], and V40 < 28–37% [150]. Grade ≥ 2 hematological toxicity was linked to increased BM volume receiving low doses, as V10 ≥ or < 90% [124].

A significant relationship emerged between the dose received by pelvic bone and nadirs of blood cells, including white blood cells, absolute neutrophil count, hemoglobin, and platelets [151]. Only V10 and V20 were significantly correlated with hemoglobin nadirs, while no dosimetric parameters were associated with platelets nadirs [124]. In cervical cancer patients who were treated with CRT, Elicin et al. found the volume of BM and aBM exposed to low doses RT were associated with white blood cells decrease. In particular, aBM V30 correlated with reduced aBM SUV and impacted the white blood cells count three months after treatment and during late follow-up [152].

In patients with cervical cancer who had no lymph node metastasis detected during surgery or by preoperative imaging, and met the criteria, reduced-volume pelvic RT, rather than whole pelvis RT, relieved acute and late radiation damage, especially myelosuppression. With a decreased CTV and significantly lower V10 and V20, reduced-volume pelvic RT did not affect long-term survival. Compared with whole pelvis RT the incidence of decreased hemoglobin associated with ≥ grade 3 thrombocytopenia toxicity was significantly reduced (*p* < 0.05) [153].

#### Treatment

During CRT, routine blood and biochemistry investigations are indicated. Myelosuppression, which can increase infection and hospitalization rates may require transfusions and administration of growth factors. It is also linked with treatment interruptions that significantly worsen outcomes [123, 125, 151].

#### Follow-up

Slow immune recovery and abnormal white blood cells count at three months post-treatment and/or at the last follow-up, underline the need to lower the incidence of hematological toxicity [152]. Low lymphocyte counts persisting for one year after RT [154] might be associated with a higher risk of decreased survival. Patients with hematological toxicity should be evaluated by a multidisciplinary team, including a hematologist. Routine analysis should include blood and biochemistry tests other than CT Scan, USG abdomen, ECG, and chest X-ray.

Summary of evidences is shown in Table 2.

### Vaginal Toxicity

#### Incidence and etiopathogenesis

Little attention is paid to vaginal toxicity and the ensuing sexual complications that women may experience after RT. In cervical cancer patients a systematic review reported more sexual dysfunction and vaginal toxicity after RT. [155]. Modifications in sexuality were due not only to physical and treatment-linked factors, but also to physiological and social causes [155]. Vaginal atrophy in up to 50–60% of women [156] affects sexuality and sexual functioning with a notable impact on QoL [157]. RT-related vaginal morbidity is mainly due to vaginal mucosa inflammation that is linked to microcirculatory alterations, leading to atrophy, telangiectasia, reduced lubrication and finally adhesions, fibrosis, vaginal stenosis and shortening.

**Fig. 6:**
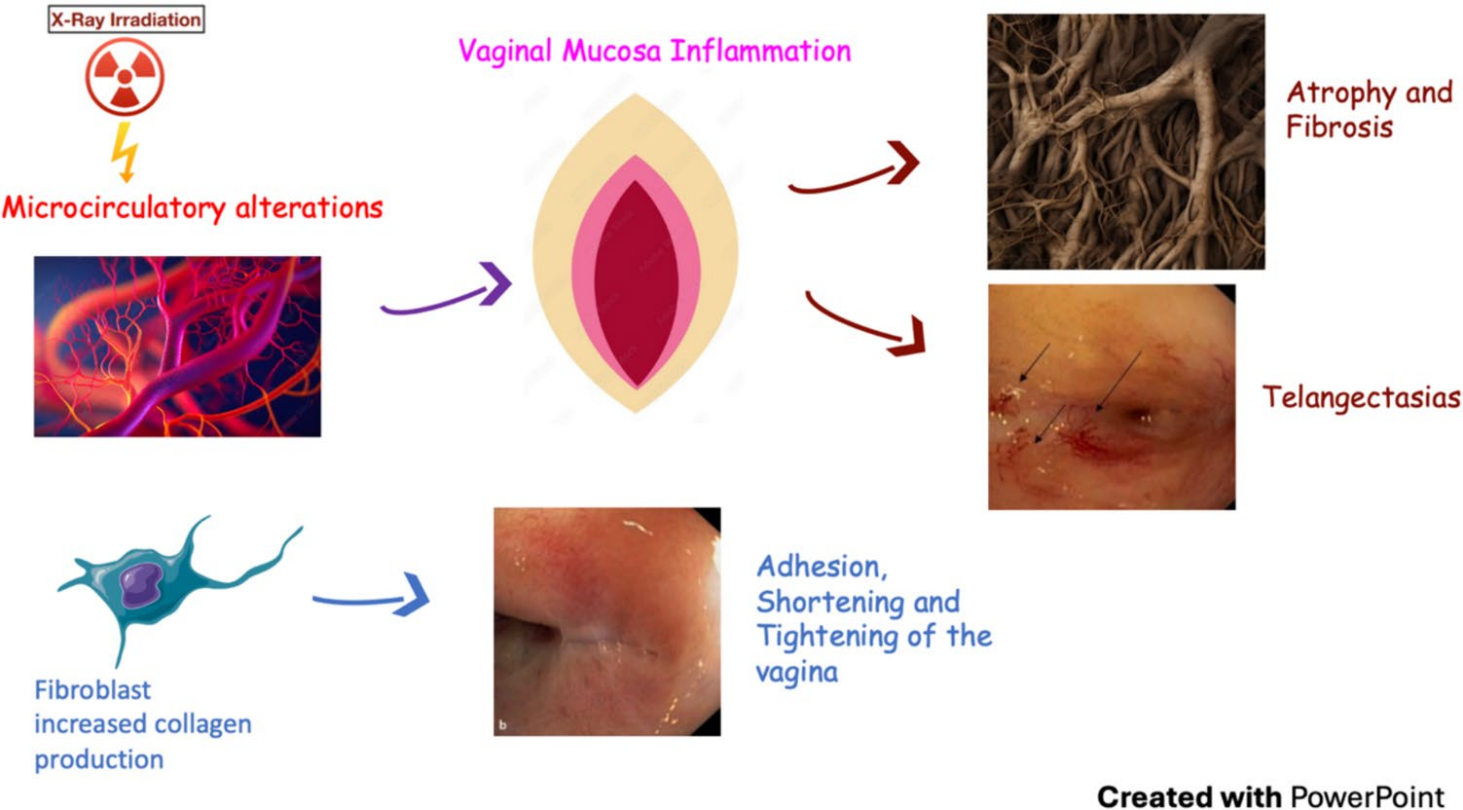
Etiopathogenesis of vaginal toxicity (K. Kirchheiner et al.Strahlenther Onkol 2012 · 188:1010–1019 https://doi.org/10.1007/s00066-012-0222-0; Fibroblast from: Smart servier medical art; Other images from: Adobe stock)

A 29% probability of grade 2 or more vaginal morbidity through the first two years after treatment was reported, with 22% actuarial probability of vaginal stenosis at 2 years [158]. Very few studies described vaginal toxicity as a Patient Reported Outcome (PRO). As assessed by PRO questionnaires, a 3-year rate of 29% vaginal dryness was reported in women treated with pelvic RT [159]. Etiopathogenesis of vaginal toxicity is shown in Fig. 6.

#### Prevention

Two dosimetric studies [158, 160] showed that improving RT techniques could prevent vaginal toxicity. Vaginal dose de-escalation at EBRT with IMRT as well as at IR is expected to reduce vaginal morbidity and thus help prevent sexual dysfunction [161]. According to data on the dose-response relationship [162], de-escalating the dose to the ICRU rectovaginal point from 75 to 65 Gy reduced grade 2 or more vaginal stenosis by 7%. Targeting multiple vaginal points gives an overview of the dose to the different parts of the vagina and appeared to be a valid strategy for reducing the dose to the vagina and correlating it to clinical outcomes [163]. In particular, doses < 50 Gy to the posterior inferior border of the pubic symphysis with EBRT + BT were associated with a lower risk of vaginal stenosis (44% incidence of grade ≥ 2 vaginal stenosis at five years *vs* 26% and 12% for patients receiving 15–50 Gy and < 15 Gy, respectively). Using 3D IR volumetric planning rather than non-volumetric point-based planning, grade 2 vaginal toxicity was significantly reduced (0% *vs* 27%) [160]. With a vaginal mucosa dose of under 140% of the fractional IR dose (corresponding to a total EQD2 of 85 Gy), the dose to the ICRU rectovaginal point was reduced from 69 to 64 Gy (*p* < 0.001) and the dose to the vaginal surface dropped from 266 to 137 Gy; the D90 HR-CTV dose was not significantly different. Overall, these changes significantly reduced vaginal toxicity more than the non-vaginal dose de-escalated plan [158].

The gonadal function might be preserved in selected cases. Ovarian preservation with IMRT is technically challenging, due to poor ovary visualization at CT planning and high oocyte radiosensitivity. Indeed, sterilization is predicted in 5 and 50% of women whose ovaries receive 2–3 Gy and 6–12 Gy, respectively [164]. Ovarian transposition and ovarian tissue preservation, as cryopreservation and transplantation, are not widely used techniques [165], but may prevent the onset of menopause, particularly in selected young cervical cancer patients. Still under evaluation are graft size, duration of the restored function according to the site of transplantation and the therapeutic modalities to reduce the risk of tumor recurrence. There is consistent evidence that heterotopic transplantation of ovarian tissue restored ovarian function for 4–5 years [165]. A recent review [166] reported that 98% of participants had restoration of ovarian function with a first ovarian transplantation.

#### Treatment

Topical application of hyaluronic acid, along with vitamin E and A [9, 167–170] prevented acute and late vaginal toxicities thanks to their role in cellular differentiation, keratinocyte proliferation, antioxidative properties and support to the extracellular matrix of the vaginal epithelium [167, 168]. They reduced dyspareunia, vaginal mucosal inflammation, vaginal dryness, bleeding, fibrosis and cellular atypia. Regular use of vaginal moisturizers to hydrate the vaginal mucosa and lubricants to minimize dryness and pain during sexual practice is indicated. Further studies are needed to confirm whether local application of mitomycin C prevents vaginal vault narrowing after treatment, as fewer vaginal adhesions and vaginal vault fibrotic changes were reported than in a control group [171].

Toxicity, deriving from hypoestrogenism, includes the genitourinary menopause syndrome, i.e., the set of vulvovaginal signs and symptoms, involving changes in the major/minor lips, clitoris, vestibule, vagina, urethra and bladder [172].

Hormone replacement therapy (HRT), as administered in diverse formulations, effectively treats genitourinary menopause syndrome [173] and is useful in managing post-RT menopausal symptoms [165]. Despite the few studies, systemic or local estrogen therapy is a valid option for acute RT-related changes and preventing the development of later vaginal complications, thanks to its direct effect on epithelial regeneration and anti-inflammatory properties. Vaginal estrogens reduce superficial dyspareunia [9] and relieve urogenital symptoms related to vaginal atrophy and are safe in cervical cancer patients because of minimal systemic absorption through the atrophic mucosa [165]. Although estrogen and progesterone receptors are expressed in 39% and 33% of cervical adenocarcinomas, HRT was not shown to significantly influence disease-free and overall survival [174]. In post-treatment menopausal cervical cancer patients, low compliance rates with HRT were reported partly due to a lack of awareness of its benefits by patients and physicians and partly because clinicians rarely prescribed HRT appropriately, fearing second malignancies such as breast and endometrial carcinoma [175]. However, estrogen-only HRT is not advised in this population, due to the risk of secondary endometrial cancer as residual function persisting after high-dose RT ends were reported [176]; while, some evidence suggested that in women undergoing a premature menopause HRT was not associated with increased breast cancer risk as long as its use continued until the age of the natural menopause [177]. No relationship emerged between HRT usage and the risk of endometrial cancer recurrence [178].

Pelvic floor muscle exercises help relieve vaginal pain and enhance clitoral blood flow, thus promoting better sexual function. Pelvic floor muscle training, alone or in combination with other treatments, seemed effective, even though more studies are needed [179].

Laser therapy was described as promising in the management of vaginal atrophy after RT as intravaginal CO_2_ laser was associated with a gradual increase in vaginal length [180].

There is no consensus on the use of vaginal dilators. Even though some authors suggest they prevent the onset and worsening of vaginal stenosis [9, 181], a systematic review [182] concluded that evidence was insufficient to recommend them, and that dilation was associated with rectovaginal fistulae and psychological consequences. Despite these findings, vaginal dilators are commonly accepted as a strategy for preventing vaginal stenosis [183]. Furthermore, their long-term use is indicated to reduce G2 late vaginal stenosis in 3D-vaginal cuff IR [184] but poor compliance might underlie minimal improvement in vaginal symptoms [185].

#### Follow-up

During follow-up visits, attention should be reserved for vaginal and sexual symptoms reported by the patients and active interventions by a multi-specialist team should be undertaken, if possible.

Summary of evidences is shown in Table 2.

## Conclusions and recommendations

Treatment of gynecological cancers may have an important impact on women’s overall health and QoL. Other than the psychological aspect linked to the diagnosis of cancer [186] patients may experience a wide range of side effects due to the multi-modal therapeutic approach which includes surgery, CHT, RT and IR. RT alone or combined with CHT as adjuvant or definitive treatment plays a crucial role in the treatment of gynecological cancers and achieves better outcomes and long-term survival of patients. However, the occurrence of acute and late side effects related to pelvic RT can negatively impact overall outcomes and patients’ QoL [187, 188].

This position paper, conceived in the AIRO Gyn Group, aimed at providing radiation oncologists with a succinct, but comprehensive view of RT-related toxicities in gynecological cancers. Aims were not only to describe the incidence and pathogenesis of specific toxicities but also, above all, to disseminate evidence for the prevention and treatment of such treatment-related side effects [3]. The ultimate goal was to provide radiation oncologists involved in gynecological cancer treatment with a practical guide to preventing, recognizing and managing specific side effects and their complications., as is required in a global approach to the patients.

Since there are no standard guidelines for narrative reviews, we decided to search PubMed, one of the largest free-access biomedical databases. We started our analysis with the year 2005, when IMRT for gynecologic tumors became standard in routine clinical practice in most Radiation Oncology Centers [189].

In our opinion, prevention of toxicity should aim at improving the therapeutic index of RT treatment, possibly by adopting IMRT/VMAT, Tomotherapy along with IGRT, which reduce the occurrence and severity of toxicity [190, 191]. Treatment planning should be done with great care, following guidelines, indications and dose constraints for OARs even though, unfortunately, dose constraints are not standardized for each specific OAR. Furthermore, to prevent the onset of toxicity, and/or reduce its severity before, during and after RT, knowledge of patient and disease features aid radiation oncologists in prescribing drugs and non-pharmacological interventions.

Moreover, patients should be carefully informed and trained if a particular preparation is required during RT treatment to avoid side effects, i.e., bladder filling or dietary recommendations if indicated. During RT treatment, patients should be followed with routine visits to early assess the occurrence and grade of toxicities, reported and graded by specific scales [4–6]. At present it is unknown if one specific scale is better than others in assessing RT-related adverse events [192]. The administration of questionnaires as PRO might be useful to recognize and prevent acute toxicity, as suggested by Chan et al. [193]. If needed, pharmacological therapy should be prescribed along with eventual replanning.

Long-term follow-up is needed to investigate not only the clinical outcome of the disease, but the occurrence of late RT-induced toxicity. Management of late toxicity can require a multidisciplinary approach and interventions should be based on shared decisions.

New evidences suggest other fields of research and interventions. Recent studies focused on the role of gut microbiome in determining gastrointestinal side effects [1, 114] and possibly treatment outcomes, indicating the need for attention to this aspect during RT. Bone health in menopausal women should not be overlooked, as bone toxicity negatively affects patients QoL. Lastly, sexual problems in women undergoing treatment for gynecological cancer have been investigated more recently [155] and the real occurrence is underestimated, as PRO revealed that patients did not respond to these specific questions [193]. Patients needing RT should be fully informed about sexual dysfunctions linked to treatment and approaches for reducing discomfort [155].

Therefore, RT techniques advance, respect for OAR constraints, knowledge of causes and treatment options for RT side effects along with patient care can guide radiation oncologists to offer the best RT modalities and support women during treatment and follow-up.

Finally, well-designed, specific investigations are needed to answer the not yet solved problems in order to improve the quality of treatment delivered to patients who will receive radiation therapy for gynecological cancers.
